# Robotic ambulatory colorectal resections: a systematic review

**DOI:** 10.1007/s11701-024-01961-3

**Published:** 2024-05-07

**Authors:** Joachim Cheng En Ho, Aryan Raj Goel, Adriel Heilong Fung, Irshad Shaikh, Muhammad Rafaih Iqbal

**Affiliations:** 1 UCL Medical School, Faculty of Medical Sciences, London, UK; 2 Norfolk and Norwich University Hospitals NHS Foundation Trust, Norwich, UK; 3 University of East Anglia, Norwich, UK

**Keywords:** Robotic surgery, Minimally invasive surgery, Ambulatory, Colorectal surgery

## Abstract

Colorectal surgery has progressed greatly via minimally invasive techniques, laparoscopic and robotic. With the advent of ERAS protocols, patient recovery times have greatly shortened, allowing for same day discharges (SDD). Although SDD have been explored through laparoscopic colectomy reviews, no reviews surrounding robotic ambulatory colorectal resections (RACrR) exist to date. A systematic search was carried out across three databases and internet searches. Data were selected and extracted by two independent reviewers. Inclusion criteria included robotic colorectal resections with a length of hospital stay of less than one day or 24 h. 4 studies comprising 136 patients were retrieved. 56% of patients were female and were aged between 21 and 89 years. Main surgery indications were colorectal cancer and recurrent sigmoid diverticulitis (43% each). Most patients had low anterior resections (48%). Overall, there was a 4% complication rate postoperatively, with only 1 patient requiring readmission due to postoperative urinary retention (< 1%). Patient selection criteria involved ASA score cut-offs, nutritional status, and specific health conditions. Protocols employed shared similarities including ERAS education, transabdominal plane blocks, early removal of urinary catheters, an opioid-sparing regime, and encouraged early oral intake and ambulation prior to discharge. All 4 studies had various follow-up methods involving telemedicine, face-to-face consultations, and virtual ward teams. RACrRs is safe and feasible in a highly specific patient population; however, further high-quality studies with larger sample sizes are needed to draw more significant conclusions. Several limitations included small sample size and the potential of recall bias due to retrospective nature of 2 studies.

## Introduction

The enhanced recovery after surgery (ERAS) model was first incepted in 1997 [1] with the aim of shortening the length of hospital stay and, therefore, lowering the cost of patient care. This model is made possible through optimizing care for patients through a preoperative, perioperative, and postoperative phase where every aspect including patient education, analgesia, fluid management, minimally invasive techniques, nutrition, and mobilization are planned for prior to the procedure itself [2]. Since then, many different pathways have been created for various procedures in their respective specialties. In colorectal surgery, this pathway has been developed for colonic and rectal resections, allowing for reductions in postoperative complications and length of hospital stay [2].

Recently, the idea of ambulatory colectomy has surfaced, where patients are discharged on the same day or within 24 h of colorectal resection. Levy et al. [3] were among the first few investigating the outcomes of ambulatory colectomies. Since then, there have been other case studies looking into its feasibility particularly through the use of laparoscopic techniques [4–11]. These studies [4–11] have been included in 2 reviews, a systematic review [12] and a scoping review [13]. Both these reviews concluded that ambulatory colectomies appear to be safe and feasible, but only in select patients where special attention was given to postoperative care. However, Siragusa et al. [12] recognized that there could be a selection bias due to the highly selective criteria for ambulatory patients operated on by highly skilled surgeons. In turn, the low rate of certain complications such as anastomotic leak and ileus [12] may not reflect reality.

The research on ambulatory colectomies thus far has been carried out using minimally invasive techniques such as laparoscopy as it enables shorter length of hospital stay [14] and is associated with a lower 30-day mortality [15]. However, robotic surgery has started to gain traction in colorectal surgery. The da Vinci system (Intuitive Surgical, Sunnyvale, CA, USA) has been at the forefront of robotic surgeries. The system has been utilized in various colorectal procedures since its FDA approval in 2000. With regards to colorectal resections, robotic techniques have been practiced since 2001 and has even been proven to be as effective and safe as its laparoscopic counterpart. Based on the current research, robotic surgery further shortens the length of hospital stay compared to laparoscopic surgery [16–18].

Currently, there is a reluctance of adopting robotic surgery particularly due to the high costs. This is often attributed to a prolonged operative time [19–21]. However, this may soon change since Vu et al. [22] have shown that operative times of robotic ambulatory colectomies have been decreasing significantly (*p* < 0.001) from 2016 to 2022. Furthermore, Ferri et al. [23] have shown that robotic right-sided colectomies are equally as cost effective as their laparoscopic counterpart and even demonstrated greater improvements in quality of life favoring the robotic group. Given the rapid innovation in robotics, the purpose of this review is to identify and summarize the findings of existing studies on the outcomes and protocols utilized in robotic ambulatory colorectal resections (RACrR).

## Methods

This study is a systematic review carried out in agreement with the preferred reporting items for systematic reviews and meta-analyses (PRISMA) guidelines. This review was registered with PROSPERO in January 2024 (CRD42024508267).

### Search strategy

Searches were carried out on 3 separate databases, Cochrane Central Register for Controlled Trials, PubMed, and Web of Science for articles published up to 10th January 2024. Keywords used are shown in Table 1. MeSH descriptor terms were only used in Pubmed and Cochrane Central Register for Controlled Trials. Alongside this, searches were performed on web browsers; Google, DuckDuckGo, and Bing with the terms “Ambulatory Robotic Col*”. The first 15 search results were looked at to see if any articles with a DOI could be included in our search that hadn’t already appeared within the database searches.

**Table 1:** Keyword search

No	Query
1	Colorectal surg* OR colorectal resection* OR rectal surg* OR colon surg* OR colectom* OR bowel resection* OR bowel surg*
*2*	MeSH descriptor: [colorectal surgery]
*3*	MeSH descriptor: [colectomy]
4	#1 or #2 or #3
5	Computer-aided surg* or image guided surg* or surg* navigat* or robot* assisted* surg* or robot* enhanced surg* or robot* enhanced procedure*
*6*	MeSH descriptor: [Robotic surgical procedures]
*7*	MeSH descriptor: [Surgery, Computer-assisted]
8	#5 or #6 or #7
9	Day case or day procedure or ambulatory or outpatient*
10	(same day OR 24 OR 23) AND (discharg* OR procedure* OR case*)
*11*	MeSH descriptor: [Ambulatory Surgical Procedures]
*12*	MeSH descriptor: [ambulatory care]
*13*	MeSH descriptor: [outpatients]
14	#9 OR #10 OR #11 OR #12 OR #13
15	#4 and #8 and #14

The restrictions applied to the database search included: limit to English-only articles and humans. Where possible, review articles were excluded when filters could be applied. Articles were then screened based on title and abstract, before another round of screening through full text.

### Inclusion criteria

Articles that included robotic colorectal resection, and length of hospital stay of less than 1 day or 24 h.

### Exclusion criteria

1. Articles where data on patients whose length of hospital stay < 24 h could not be extracted
2. Length of hospital stay was not explicitly mentioned to be < 24 h (used mean or only showed interquartile range)
3. Procedures not relating to colorectal resection (transrectal and hernia procedures were excluded)
4. Articles which used the same patient population (the more recent article was selected to be included).

### Data collection and analysis

Each full-text article was screened by 2 authors independently (J.C.E.H. and A.R.G.) for the following information: title, first author, study design, year of publication, country, number of patients (sample size), and outcome measurements. If there were any disagreements, they were discussed. A senior author (M.R.I.) was consulted if there were persisting disagreements.

### Outcome measurements

Primary outcomes included 30-day post-operative complications and readmissions. Secondary outcomes were the reoperation rate, mean estimated blood loss, mean operative time, and unscheduled hospital visits.

Patients baseline characteristics were also obtained. These were analyzed and included age, sex, Body Mass Index (BMI), American Society of Anaesthesiologists (ASA) score, type of surgery, indication of surgery, and patient acceptance rate (which is the proportion of patients who accepted the offer of an ambulatory robotic colorectal procedure). The protocols of each of the robotic ambulatory colorectal procedures were analyzed and retrieved. This comprised the patient selection criteria, the pre- and postoperative management, which also included the discharge plan, discharge criteria, analgesic measures, and safety netting.

### Quality assessment

The quality of the study was assessed using the Joanna Briggs Institute (JBI) Critical Appraisal tool [24]. Studies were assessed using this tool by 2 authors (J.C.E.H. and A.R.G.) independently. This involved a series of yes/no questions to assess the trustworthiness, results, and relevance of existing papers. The appraisal tool queried the standardization of outcome measurements, inclusion of participants, reliability of participant characteristics reporting, and follow-up methods. Any disagreements in quality assessment and risk of bias were resolved by discussion involving the senior author (M.R.I.).

## Results

### Systematic search results

The search through the 3 databases yielded a total of 1403 articles. The automated filter functions removed 7 studies due to the limitations of English language and human studies. 47 duplicate articles were then removed. 6 other articles were obtained from other sources. A total of 1349 articles were then screened for relevancy based on title and abstract, from which 1320 articles were removed due to neither the title nor abstract meeting our inclusion criteria, 21 of these articles were removed due to the articles specifically being animal-based studies. 29 articles were screened based on full text and articles that did not meet the inclusion criteria were removed. This left 3 studies, of which 1 was removed due to sharing the same population sample as a more recent paper by the same group of researchers. Using the other method for completion of our search described in the above methods, we obtained 6 records that were not yielded in our database search, 4 were found to be duplicates, thus leaving additional 2 studies to be included. Finally, a total of 4 studies were selected to be included in the systematic review.

**Fig. 1:**
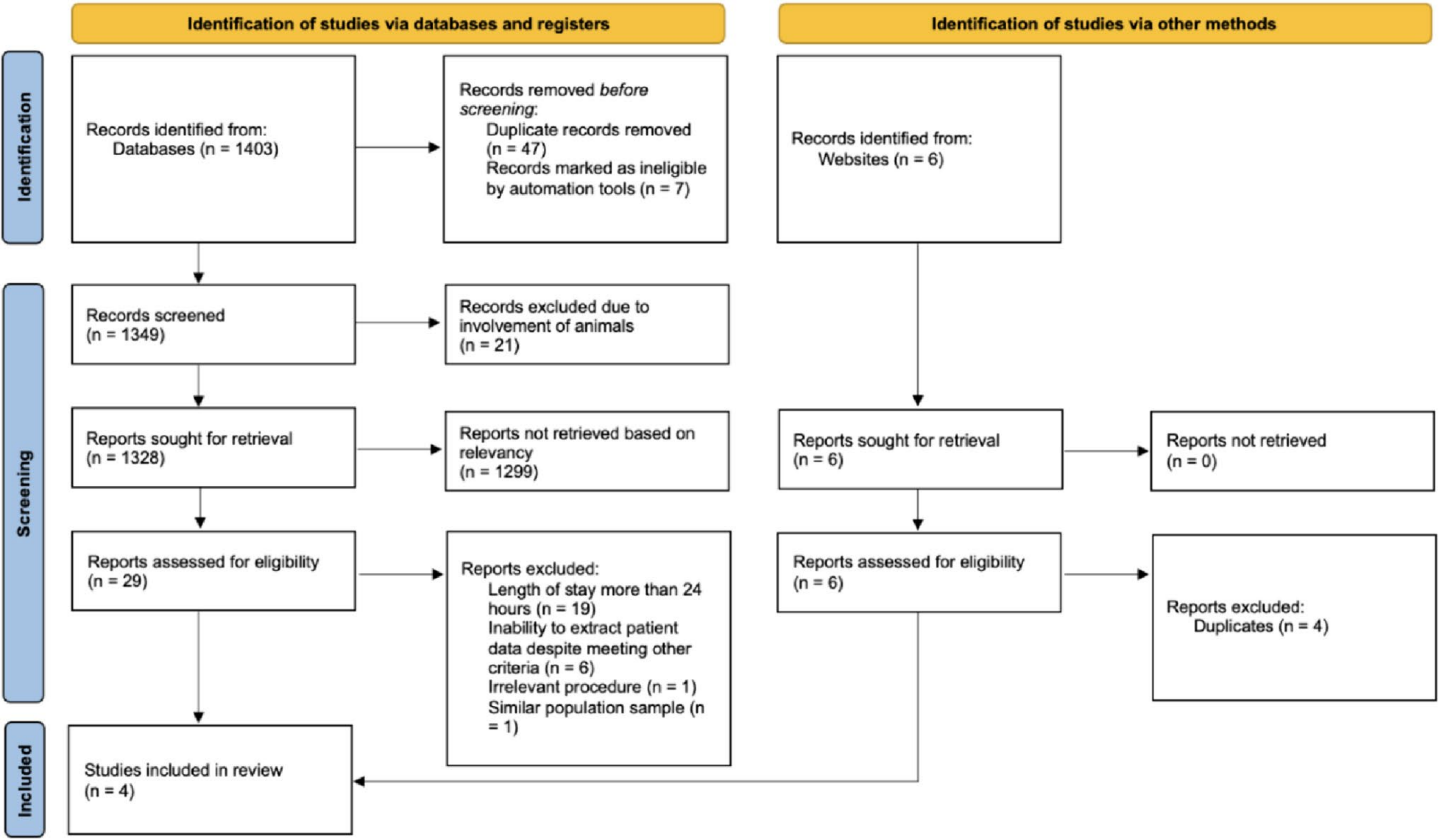
Systematic search results using PRISMA flow diagram

The systematic search in accordance with PRISMA guidelines is shown in Fig. 1.

### Study characteristics

A total of 4 articles were published from 2021 to 2023. These include 2 retrospective [25, 26] and 2 prospective [27, 28] articles. The number of patients ranged from 3 [28] to 115 [26], with a total of 136 patients. 3 of the studies [25–27] were carried out in United States of America (USA), while only 1 [28] was conducted in the United Kingdom (UK) (Table 2).

**Table 2:** Study characteristics

Study	Year of publication	Country	Study design	Number of patients
Campbell et al. [25]	2021	USA	Retrospective	7
Curfman et al. [26]	2022	USA	Retrospective	115
Bowman et al. [27]	2023	USA	Prospective	11
Hamed et al. [28]	2023	UK	Prospective	3

### Patient characteristics

Among 136 patients, 60 males and 76 females (56% female). The mean age was 58 ( range 21–89 years). BMI was only reported in 2 studies [27, 28] and had a mean of 25 and 29 kg/m^2^, with a cumulative range of 21–39 kg/m^2^. The ASA scores were reported in 3 studies, where 2 of the studies [27, 28] reported that all patients had an ASA II grade, while Curfman et al. [26] limited the patient population to a maximum of ASA III. The types of surgery carried out were as follows (136 patients): 48% in low anterior resections, 21% in right colectomies, 11% in sigmoidectomies, 7% in cecectomies, 2% in transverse colectomies, proctectomies, left colectomies and high anterior resections each, and 1% in Hartmann’s reversal procedure. Indication for surgery was reported in 3 studies [25, 27, 28] (21 patients): 43% in colorectal cancer, 43% in recurrent sigmoid diverticulitis, 5% in cecal volvulus, 5% in Crohn’s disease, and 5% in an unknown indication for Hartmann’s reversal procedure. Bowman et al. [27] (11 patients) was the only study to report a patient acceptance rate, of 73% (Table 3).

**Table 3:** Patient characteristics

Study	Number of patients	Age, mean (range, years)	Sex (*n*, %)	BMI, mean (range, kg/m^2^)	ASA score (*n*, %)	Type of surgery (*n*, %)	Indication for surgery (*n*, %)	Patient acceptance rate (*n*, %)
Campbell et al. [25]	7	57 (39–77)	M 4 (57%) F 3 (43%)	N/A	N/A	LAR 4 (57%) RH 2 (29%) HR 1 (14%)	Colorectal cancer 6 (86%) Unknown 1 (14%)	N/A
Curfman et al. [26]	115	59 (21–89)	M 48 (42%) F 67 (58%)	N/A	< ASA IV 115 (100%)*	LAR 61 (53%) RH 25 (22%) CC 9 (8%) SD 6 (5%) PH 5 (4%) LH 3 (3%) TC 3 (3%) PT 3 (3%)	N/A	N/A
Bowman et al. [27]	11	43 (34–62)	M 6 (55%) F 5 (45%)	29 (21–39)	ASA II 11 (100%)	SD 9 (82%) RH 2 (18%)	Diverticulitis 9 (82%) Cecal volvulus 1 (9%) Crohn’s disease 1 (9%)	11/15 73%
Hamed et al. [28]	3	57 (51–64)	M 2 (67%) F 1 (33%)	25 (23–28)	ASA II 3 100%	HAR 3 100%	Colorectal cancer 3 (100%)	N/A

*N/A* not available, *M* male, *F* female, *LAR* low anterior resection, *RH* right hemicolectomy, *HR* Hartmann’s reversal, *CC* cecectomy, *SD* sigmoidectomy, *PH* parastomal hernia, *LH* left hemicolectomy, *TC* transverse colectomy, *PT* proctectomy, *HAR* high anterior resection

^*^Value obtained from Patient inclusion/exclusion criteria

### Outcomes

In terms of the primary outcomes (Table 4), all 4 studies reported data on 30-day complications and readmissions. 2 studies [27, 28] (14 patients) reported no complications, while the other 2 studies [25, 26] (122 patients) reported 14% and 3%, respectively, an overall of 4%. Specific complications include nausea and abdominal pain (1%), vomiting (< 1%), urinary retention (< 1%), leg pain (< 1%), diarrhea (< 1). Only 1 patient (< 1%) required to be readmitted because of postoperative urinary retention in the study of Curfman et al. [26] (115 patients), the other 3 studies [25, 27, 28] (21 patients) reported no postoperative readmissions.

**Table 4:** Primary and secondary outcomes

Study	30-day complications (*n*, %)	30-day readmissions (*n*, %)	Reoperation rate (*n*, %)	Mean EBL (mL)	Mean operative time (mins)	Unscheduled hospital visits (*n*, %)
Campbell et al. [25]	1/7 (14%)	0	0	N/A	N/A	2/7 (29%)
Curfman et al. [26]	5/115 (3%)	1/115 (< 1%)	0	N/A	149	5/115 (3%)
Bowman et al. [27]	0	0	0	30	132	0
Hamed et al. [28]	0	0	0	N/A	N/A	0

*N/A* not available, *EBL* estimated blood loss

There were no reoperations in all 4 studies, while mean estimated blood loss (EBL) was reported in 1 study [27], 30 mL. Mean operative times were reported in 2 studies [26, 27] (126 patients), as 149 and 132 min, respectively. Unscheduled hospital visits occurred in 5% of all 4 studies, which was 1 patient more than the complications during the 30-day postoperative period. This patient, in the study of Campbell et al. [25], returned for teaching on ostomy care and fluid management.

### Protocol

All 4 studies were included in these sections.

### Patient selection criteria

The extent of colorectal resection (partial or total) was used as a criterion in all 4 studies [25–28], with total colectomies being excluded. Age was used as a cut-off in 2 studies [25, 28] (75 and 80 years as the upper limit, respectively), while ASA scores were indicated in 3 studies [26–28], 2 of which [27, 28] limited them to a score of ASA I–II. All studies reported on the necessity of having a good support system postoperatively. Curfman et al. [26] even highlighted the need for the patient and their support team to understand the procedure and perioperative management. 3 studies [26–28] excluded patients who had a history or were currently on anticoagulation or antiplatelet medication. Nutritional status was taken into consideration in 2 studies [25, 26], while BMI was only indicated in 1 study [28]. 3 studies [26–28] reported on the exclusion of patients who needed the creation of a stoma, 2 of which [26, 28] also specified the exclusion of procedures involving conversions to open surgery. Specific health conditions posing as contraindications for RACrR included cardiac conditions [25, 26], diabetes mellitus [26, 28], previous pelvic radiotherapy [28], frailty score ≥ 3 [25], or complex frailty [28] (Table 5).

**Table 5:** Inclusion and exclusion criteria for patient selection

Study	Inclusion criteria	Exclusion criteria
Campbell et al. [25]	• Partial colectomies • Candidate for laparoscopy • ≤ 2 simple comorbidities • Age ≤ 75 years old • Adequate nutritional status	• > 2 simple comorbidities • History of severe cardiac condition, i.e., arrhythmia, heart failure) • Frailty score ≥ 3 • Poor home support system
Curfman et al. [26]	• Means for physician contact • Adequate support team consists of someone who will be with the patient after discharge and be able to care of them postoperatively • Understanding of the procedure and perioperative course by patient and their support team	• Emergent surgery, total abdominal colectomy, ostomy creation, major prior abdominal surgeries, major uncontrolled comorbidities (such as diabetes mellitus or cardiopulmonary disease), history of anticoagulation or antiplatelet use, malnutrition, • ASA IV • Intraoperative complications and conversions to open surgery
Bowman et al. [27]	• ASA I–II • Must have a chaperone at home for 3 days post-operatively • Must be compliant with low risk for lost to follow-up • Robotic surgery for partial colectomy or low anterior resection without ileostomy	• Total colectomy and requirement of ileostomy • Patients on pre-operative anticoagulation
Hamed et al. [28]	• < 80 years old • BMI < 35 • ASA I–II • Minimal or no previous abdominal surgery • Elective robotic colonic resections (left sided, right sided) for colorectal cancer • Have a social support network upon discharge from hospital	• Conversion to open procedure and creation of a stoma • Diabetes mellitus, previous pelvic radiotherapy, complex frailty requiring multi modal interventions • Living alone upon discharge, being a nursing or residential home resident, inability to take medication independently or with help from relatives/carers • Cognitive impairment limiting the ability to use home monitoring or undertake telephone/video calls or follow-up on escalation pathway • On anticoagulation or antiplatelets

*N/A* not available, *ASA* American Society of Anesthesiologists score, *BMI* Body Mass Index

### Perioperative management and discharge criteria

All 4 studies reported preoperative, intraoperative, and postoperative management protocols.

In preoperative management, 3 of the studies [25, 26, 28] involved patient education on the procedure itself: risks and benefits [26, 28], dietary advice [26, 28], and potential complications including prolonged stay [26] and pathway escalations [28]. Bowel preparation was documented in 2 studies [26, 27], and preoperative analgesic use, specifically acetaminophen, gabapentin and low-dose opioids were documented in 2 studies [25, 27]. To reduce postoperative ileus, alvimopan was given in 2 studies [25, 27] preoperatively. 2 studies [25, 27] also documented the use of pharmacologic thromboprophylaxis preoperatively.

Intraoperatively, a transverse abdominal plane (TAP) block employed for pain management was documented in 3 studies [25–27]. 2 studies [25, 27] reported that all cases incorporated intracorporeal anastomoses, while 2 studies [26, 27] also reported on avoiding opiate use where possible, as well as the use of a multimodal anesthetic approach [26, 27]. The avoidance of intra-abdominal drains was stated in 2 studies [26, 28], in which 1 study [28] also avoided the use of nasogastric (NG) tubes. Fluid management was a focus in 3 studies [26–28], where 1 study [27] had a goal-directed fluid therapy which limited the intravenous (IV) fluids to less than 2500 mL of isolute. 3 studies [26–28] advocated the early removal of urinary catheters, with the 1 other study [25] encouraging the removal of catheters prior to being discharged (patients were discharged with a leg bag if they were still unable to void). 2 studies [26, 28] highlighted the use of minimally invasive techniques, in which Curfman et al. [26] further specified the use of sweeping methods for manipulation instead of regular tissue grabbing.

In the postoperative management segment, all 4 studies reported the use of analgesics, specifically acetaminophen (paracetamol), naproxen, gabapentin and tramadol. Hydromorphone was given only for severe pain in 1 study [26]. The early initiation of oral intake was reported in 3 studies [25, 26, 28], 1 of which [25] specified a fluid liquid diet. Early mobilization was encouraged in 2 studies [25, 28], while pharmacologic thromboprophylaxis use was reported in 3 studies [25, 27, 28]. 1 study [26] reported the use of incentive spirometer education and use, 1 [27] abdominal binder use for 5 days, 1 [27] alternating ice pack/heating pack, and 1 [26] anti-nausea medication (usually ondansetron).

In terms of follow-ups, all reported various follow-up protocols. All the follow-ups arranged from postoperative day 1 to day 4 were carried out remotely through telemedicine [25–28]. 3 studies [26–28] specified the involvement of colorectal surgeons in the follow-up period. In 2 studies [26, 27], telemedicine further involved physician associates (PAs), whereas 1 study [28] utilized a virtual ward team in telemedicine. In Hamed et al.’s study [28], observations including vital signs and urine output were recorded through remote monitoring equipment.

2 studies [26, 27] documented their discharge criteria, where both studies [26, 27] required patients to be able to void, have little to no nausea, and controlled pain prior to discharge. Specific requirements also included tolerating oral intake [26], EBL of less than 150 mL [27], case duration less than 4 h [28], hemodynamically stable [27], and a post-anesthesia care unit (PACU) observation time of at least 3 h [27].

The perioperative (preoperative, intraoperative and postoperative) management protocols and discharge criteria are outlined in Table 6, 7 and 8. Specific follow-up protocols are detailed in Table 9.

**Table 6:** Preoperative management as part of the ambulatory robotic surgery protocols

	Campbell et al. [25]	Curfman et al. [26]	Bowman et al. [27]	Hamed et al. [28]
ERAS Education	✓	✓		✓
VTE prophylaxis	✓		✓ (heparin 5000U or enoxaparin 40 mg)	
Establishing a communication method or support network		✓ (communication method)		✓ (specialist nurse support network)
Bowel prep		✓	✓	
Alvimopan	✓	✓	✓ (12 mg)	✓
Pre-operative pain relief	✓ (acetaminophen, gabapentin, NSAIDs and low-dose narcotics)		✓ (gabapentin, acetaminophen, and tramadol)	

*ERAS* Enhanced Recovery After Surgery, *VTE* Venous Thromboembolism, *NSAIDs* Nonsteroidal anti-inflammatory drugs

**Table 7:** Intraoperative management as part of the ambulatory robotic surgery protocols

	Campbell et al. [25]	Curfman et al. [26]	Bowman et al. [27]	Hamed et al. [28]
TAP block	✓	✓ (bupivacaine or ropivacaine)	✓ (bupivacaine)	
Opioids		Avoided specifically	Avoided specifically (with benzodiazepines)	
Pain management		✓	✓ (Ketamine, lidocaine)	
NG tubes/drains		Avoided		Avoided
Urinary catheters	✓ (removal post-op if tolerated)	✓ (avoidance of continued use)	✓ (early removal)	✓ (early removal)
Intracorporeal anastomoses	✓		✓	
Fluid management	✓	✓ (decreased)	✓ (limited to less than 2500 ml)	✓

**Table 8:** Postoperative management and discharge criteria as part of the ambulatory robotic surgery protocols

	Campbell et al. [25]	Curfman et al. [26]	Bowman et al. [27]	Hamed et al. [28]
Postoperative oral pain regimen	✓	✓ (acetaminophen, naproxen and gabapentin, if needed hydromorph)	✓ (acetaminophen 600 mg every 6 h for 5 days, Tramadol 50 mg every 4 h)	✓
IV fluids	✓	✓ (no or minimal)		
Oral intake in recovery unit	✓	✓	✓	✓
VTE prophylaxis	✓ (Lovenox was arranged if needed)	✓	✓ (Enoxaparin 40 mg)	✓
Ambulation pre-discharge	✓	✓	✓	✓
Follow-up	✓ (option of in-person, outpatient or telephone + contact daily with a clinic until POD4, 2-week follow-up)	✓ (contacted by team on POD1, POD3 + seen in clinic between POD5-7)	✓ (Call at 8 pm POD0, 2 calls at 8am + 4 pm POD1, 8am POD2, 8am POD3 + clinic 2 weeks post-discharge)	✓ (Daily review on virtual ward until POD3 when formal monitoring was stopped)
Diet	✓ (full liquid POD1, then regular diet as tolerated)	✓ (able to tolerate oral intake pre-discharge)		
Recommendation to stay near hospital postoperatively	✓	✓		
Education	✓	✓		✓
Antiemetics		✓ (ondansetron promethazine and/or metoclopramide)		

*POD* postoperative day

**Table 9:** Follow-up schedules of each ambulatory robotic surgery protocol

	Campbell et al. [25]	Curfman et al. [26]	Bowman et al. [27]	Hamed et al. [28]
POD0			✓	✓
POD1	✓	✓	✓	✓
POD2	✓		✓	✓
POD3	✓	✓	✓	✓
POD4	✓			
POD5		✓*		
POD6				
POD7				
POD14	✓		✓	

*POD* postoperative day

^*^indicates that a follow-up session was scheduled between POD5 and POD7

## Discussion

The current review encompasses 4 studies [25–28] and compares the patient baseline characteristics, specific outcomes, and individual study protocols. The main findings of this review include that RACrR is a safe procedure in those that meet the strict criteria for this form of surgical practice. It needs a vigorous patient selection criteria, multilevel patient management and intense postoperative monitoring.

Generally, the studies indicated that patients were required to be ≤ ASA II which was either explicitly stated or criteria was set to comply with this classification, this was a feature in 2 studies [27, 28]. Curfman et al. [26] was an exception to this, allowing patients of up to ASA III to be included in their study based on selection criteria. However, due to the absence of patient data on ASA classification, it is difficult to conclude or estimate the proportion of ASA III in the study [26]. All 4 studies [25–28] necessitated a good support system following the procedure, whether that be a hospital provided chaperone or home support, allowing for close postoperative monitoring. In the study from Campbell et al. [25], patients were advised to stay close to the hospital postoperatively for the ease of in-person follow-ups. There was also a method of remote monitoring utilized by Hamed et al. [28], where a virtual ward team was monitoring the patients’ recovery remotely. Patient education was seen as crucial to the protocol’s success. The patients and relevant support members in the study of Curfman et al. [26] received hardcopies of education material and the level of understanding was checked using a teach-back method. Such methods of preoperative education are important and is already a proven method of reducing current ERAS protocols [33].

Siragusa et al. [12] performed a systematic review looking into ambulatory colectomies, but only consisted of laparoscopic methods. In their review, they concluded that ambulatory laparoscopic colectomies were safe and practical to be carried out but also acknowledged several limitations, such as a narrow patient selection criteria and only retrospective studies to work with. Currently, there exist multiple studies [16, 17, 29–32] suggesting that robotic colectomies result in shorter LOS compared to laparoscopic methods. Hence, it seemed necessary to evaluate the potential and feasibility of RACrR. Based on our review of 136 patients, there was a complication rate of 4%, readmission rate of < 1%, and no reoperation. There was one readmission due to urinary retention in the study of Curfman et al. Other complications were less severe and did not require readmission, which is reassuring and indicative of RACrR as a safe procedure.

In this review, there was certainly an element of heterogeneity. There were different terms given to the protocol, such as Same day discharge (SDD) protocol [26] and having outpatient major elective (HOME) robotic colon resection protocol [27]. There were also differences in methods of patient monitoring, ranging from the use of PAs, frequency and duration of postoperative monitoring periods and remote monitoring equipment. More importantly, there were different indications of surgery including cancers, inflammatory bowel diseases, and diverticular diseases. These variations would affect the type of partial colectomy carried out, influencing the outcomes of the robotic surgery itself. A more sensible approach would be to carry out randomized controlled trials on specific patient populations with a specific indication for surgery.

Despite the differences, there were many features present across all four protocols in agreement with each other. Much of these were similar or adapted from the existing ERAS guidelines in colorectal surgery [34]. The studies describe their perioperative management, in particular Curfman et al. [26] which indicated in their SDD protocol the aspects that they kept the same from current ERAS guidelines and modifications they had made. Preoperatively, the use of antibiotic bowel prep, multimodal analgesics, and alvimopan allowed for reduced surgical site infections [33], reduced opioid consumption postoperatively [35], which indirectly reduces postoperative nausea and vomiting (PONV) [34] and a shorter time to restore bowel function [36], respectively. Intraoperatively, there was once again the use of opioid sparing analgesia methods, including TAP blocks which helped further reduced the risk of PONV [37]. There were also specific minimally invasive techniques described by Curfman et al. [26] which emphasize the avoidance of excessive grasping, thereby lowering the mechanical compressive forces within the tissues [38]. The avoidance of indwelling catheters was important to the postoperative management stage as urinary catheters often pose as a barrier to early mobilization and urinary tract infections, ultimately prolonging length of hospital stay [39, 40]. Intracorporeal anastomotic techniques were used in 2 studies [25, 27], which may be more effective to be implemented into future protocols as they reduce the complication rates [41].

Postoperatively, early mobilization and oral intake was a priority. Early mobilization was aided by opioid sparing techniques and would in turn prevent prolonged bed rest, which is associated with other complications as highlighted in the ERAS recommendations [34]. Early oral intake reduces length of hospital stay and has been proven to reduce overall postoperative complications [42]. Interestingly, the use of gum-chewing to reduce ileus was not employed in the above protocols. Gum-chewing has been described in several studies [4, 9, 43] based on the review by Siragusa et al. [12]. Sham feeding, by means of gum-chewing may have some effect on reducing LOS by bowel stimulation and thus ileus prevention, and should be considered for future protocols [44, 45].

Only one of the studies was done in a UK-based site [28], thus the majority of current information is stemming from the USA which should raise questions about the applicability of this practice within the main system in the UK, the NHS. Community services are already stretched [46, 47] thus further research will need to be done to determine how much of a possibility there is for robotic ambulatory pathways in the UK population.

To our knowledge, this is the first review to investigate the outcomes and feasibility of RACrR. As our literature search only retrieved 4 studies [25–28] that met the inclusion criteria, this meant that there was a small sample size to draw conclusions from. This may have been a result of the COVID pandemic, as Bowman et al. [27] alluded to. Another limitation was the non-comparative nature of the results, thus making the results prone to selection bias. Of the 4 studies, 2 were retrospective, which will also increase the results’ susceptibility to recall bias. The current publications have no real standardization of methods of their approach nor protocol and there are no standard definitions leading to variation in their primary outcomes.

Further studies should be designed comparatively with larger sample sizes utilizing validated protocols for specific indications for surgery. As mentioned earlier, the usage of intracorporeal anastomosis may be important in reducing the complication rate, alongside the use of gum-chewing to further shorten the LOS.

## Conclusion

Based on the very limited results, our review concludes that RACrR may be considered safe and feasible for a very specific cohort of patients. Further high-quality studies looking into larger populations with specific characteristics are warranted to draw more significant conclusions.

## Data Availability

All the data generated or analyzed during this study is included in the article. Further inquiries can be directed to the corresponding author.

## References

[1] Taurchini M, Del Naja C, Tancredi A (2018) Enhanced recovery after surgery: a patient centered process. J Vis Surg. 10.21037/jovs.2018.01.20 29552522 10.21037/jovs.2018.01.20 PMC5847857

[2] Policarpo F, Cardoso V, Boligo S et al (2023) Implementation of an ERAS® Pathway in colorectal surgery in three different hospitals of the same hospital center-strategies to optimize compliance of healthcare professionals with the ERAS® protocol. Clin Nutr ESPEN 57:809–810

[3] Levy BF, Scott MJP, Fawcett WJ, Rockall TA (2009) 23-hour-stay laparoscopic colectomy. Dis Colon Rectum 52:1239–1243 19571699 10.1007/DCR.0b013e3181a0b32d

[4] Gignoux B, Gosgnach M, Lanz T et al (2019) Short-term outcomes of ambulatory colectomy for 157 consecutive patients. Ann Surg 270:317–321 29727328 10.1097/SLA.0000000000002800

[5] Gash KJ, Goede AC, Chambers W et al (2011) Laparoendoscopic single-site surgery is feasible in complex colorectal resections and could enable day case colectomy. Surg Endosc 25:835–840 20734083 10.1007/s00464-010-1275-8

[6] Brandt E, Poulsen M, Lykke J et al (2013) A minority of patients discharged within 24 hours after laparoscopic colon resection. Dan Med J 60:A4658 23809969

[7] Dobradin A, Ganji M, Alam SE, Kar PM (2013) Laparoscopic colon resections with discharge less than 24 hours. JSLS 17:198 23925012 10.4293/108680813X13654754535791 PMC3771785

[8] Studniarek A, Borsuk DJ, Kochar K et al (2021) Feasibility assessment of outpatient colorectal resections at a tertiary referral center. Int J Colorectal Dis 36:501–508 33094353 10.1007/s00384-020-03782-w

[9] Lee L, Eustache J, Baldini G et al (2022) Enhanced recovery 2.0–same day discharge with mobile app follow-up after minimally invasive colorectal surgery. Ann Surg 276:e812–e818 34091514 10.1097/SLA.0000000000004962

[10] McKenna NP, Bews KA, Shariq OA et al (2020) Is same-day and next-day discharge after laparoscopic colectomy reasonable in select patients? Dis Colon Rectum 63:1427–1435 32969886 10.1097/DCR.0000000000001729

[11] Popeskou SG, Christou N, Panteleimonitis S et al (2022) Safety and feasibility of a discharge within 23 hours after colorectal laparoscopic surgery. J Clin Med 11:5068 36078996 10.3390/jcm11175068 PMC9456718

[12] Siragusa L, Pellino G, Sensi B et al (2023) Ambulatory laparoscopic colectomies: a systematic review. Colorectal Dis. 10.1111/codi.16511 36790358 10.1111/codi.16511

[13] Tan JKH, Choe L, Lau J, Tan K-K (2022) Discharge within 24 hours following colonic surgery—a distant dream or near reality? A scoping review. Surgery. 10.1016/j.surg.2022.04.050 35840425 10.1016/j.surg.2022.04.050

[14] Reza MM, Blasco JA, Andradas E et al (2006) Systematic review of laparoscopic versus open surgery for colorectal cancer. J British Surg 93:921–928 10.1002/bjs.5430 16845692

[15] Zheng Z, Jemal A, Lin CC et al (2015) Comparative effectiveness of laparoscopy vs open colectomy among nonmetastatic colon cancer patients: an analysis using the National Cancer Data Base. J Natl Cancer Inst. 10.1093/jnci/dju491 25663688 10.1093/jnci/dju491 PMC4565531

[16] Raskin ER, Gorrepati ML, Mehendale S, Gaertner WB (2019) Robotic-assisted ileocolic resection for Crohn’s disease: outcomes from an early national experience. J Robot Surg 13:429–434 30426352 10.1007/s11701-018-0887-1

[17] McCarthy E, Gough BL, Johns MS et al (2021) A comparison of colectomy outcomes utilizing open, laparoscopic, and robotic techniques. Am Surg 87:1275–1279 33345569 10.1177/0003134820973384

[18] Clapp B, Klingsporn W, Harper B et al (2019) Utilization of laparoscopic colon surgery in the Texas inpatient public use data file. J Soc Laparoendos Surg. 10.4293/JSLS.2019.00032 10.4293/JSLS.2019.00032 PMC6708411 31488941

[19] Merola G, Sciuto A, Pirozzi F et al (2020) Is robotic right colectomy economically sustainable? A multicentre retrospective comparative study and cost analysis. Surg Endosc 34:4041–4047 31617088 10.1007/s00464-019-07193-z

[20] Petrucciani N, Sirimarco D, Nigri GR et al (2015) Robotic right colectomy: A worthwhile procedure? Results of a meta-analysis of trials comparing robotic versus laparoscopic right colectomy. J Minim Access Surg 11:22 25598595 10.4103/0972-9941.147678 PMC4290114

[21] Davis BR, Yoo AC, Moore M, Gunnarsson C (2014) Robotic-assisted versus laparoscopic colectomy: cost and clinical outcomes. JSLS 18:211 24960484 10.4293/108680813X13753907291035 PMC4035631

[22] Vu MM, Curfman KR, Blair GE et al (2023) Beyond enhanced recovery after surgery (ERAS): evolving minimally invasive colectomy from multi-day admissions to same-day discharge. American J Surg 225:826–831 10.1016/j.amjsurg.2023.01.024 36697356

[23] Ferri V, Quijano Y, Nuñez J et al (2021) Robotic-assisted right colectomy versus laparoscopic approach: case-matched study and cost-effectiveness analysis. J Robot Surg 15:115–123 32367439 10.1007/s11701-020-01084-5

[24] Munn Z, Barker TH, Moola S et al (2020) Methodological quality of case series studies: an introduction to the JBI critical appraisal tool. JBI Evid Synth 18:2127–2133 33038125 10.11124/JBISRIR-D-19-00099

[25] Campbell S, Fichera A, Thomas S, et al. (2022). Outpatient colectomy—a dream or reality? In: Baylor University Medical Center Proceedings. Taylor and Francis 10.1080/08998280.2021.1973327 PMC8682818 34970026

[26] Curfman KR, Poola AS, Blair GE et al (2023) Ambulatory colectomy: a pathway for advancing the enhanced recovery protocol. J Robot Surg 17:827–834 36334255 10.1007/s11701-022-01463-0 PMC9638390

[27] Bowman D, Proctor C, Richards K, Protyniak B (2023) Having outpatient major elective (HOME) robotic colon resection protocol: a safe approach to ambulatory colon resection. Am Surg 89:6078–6083 37470507 10.1177/00031348231189829

[28] Hamed M (2023) Feasibility of ambulatory robotic colorectal cancer surgery: the initial experience of a UK teaching hospital. Biomed J Sci Tech Res. 10.26717/BJSTR.2023.51.008173

[29] Zhu X-L, Yan P-J, Yao L et al (2019) Comparison of short-term outcomes between robotic-assisted and laparoscopic surgery in colorectal cancer. Surg Innov 26:57–65 30191755 10.1177/1553350618797822

[30] Trastulli S, Coratti A, Guarino S et al (2015) Robotic right colectomy with intracorporeal anastomosis compared with laparoscopic right colectomy with extracorporeal and intracorporeal anastomosis: a retrospective multicentre study. Surg Endosc 29:1512–1521 25303905 10.1007/s00464-014-3835-9

[31] Shiomi A, Kinugasa Y, Yamaguchi T et al (2016) Robot-assisted versus laparoscopic surgery for lower rectal cancer: the impact of visceral obesity on surgical outcomes. Int J Colorectal Dis 31:1701–1710 27599703 10.1007/s00384-016-2653-z

[32] Donlon NE, Nugent TS, Free R et al (2021) Robotic versus laparoscopic anterior resections for rectal and rectosigmoid cancer: an institutional experience. Irish J Med Sci. 10.1007/s11845-021-02625 33846946 10.1007/s11845-021-02625-z

[33] Cavallaro PM, Milch H, Savitt L et al (2018) Addition of a scripted pre-operative patient education module to an existing ERAS pathway further reduces length of stay. Am J Surg 216:652–657 30041735 10.1016/j.amjsurg.2018.07.016

[34] Gustafsson UO, Scott MJ, Hubner M et al (2019) Guidelines for perioperative care in elective colorectal surgery: enhanced recovery after surgery (ERAS®) Society recommendations: 2018. World J Surg 43:659–695 30426190 10.1007/s00268-018-4844-y

[35] Baloyiannis I, Theodorou E, Sarakatsianou C et al (2020) The effect of preemptive use of pregabalin on postoperative morphine consumption and analgesia levels after laparoscopic colorectal surgery: a controlled randomized trial. Int J Colorectal Dis 35:323–331 31863206 10.1007/s00384-019-03471-3

[36] Delaney CP, Wolff BG, Viscusi ER et al (2007) Alvimopan, for postoperative ileus following bowel resection: a pooled analysis of phase III studies. Ann Surg 245:355 17435541 10.1097/01.sla.0000232538.72458.93 PMC1877012

[37] Peltrini R, Cantoni V, Green R et al (2020) Efficacy of transversus abdominis plane (TAP) block in colorectal surgery: a systematic review and meta-analysis. Tech Coloproctol 24:787–802 32253612 10.1007/s10151-020-02206-9

[38] Barrie J, Russell L, Hood AJ et al (2018) An in vivo analysis of safe laparoscopic grasping thresholds for colorectal surgery. Surg Endosc 32:4244–4250 29602989 10.1007/s00464-018-6172-6 PMC6132882

[39] Surkan MJ, Gibson W (2018) Interventions to mobilize elderly patients and reduce length of hospital stay. Can J Cardiol 34:881–888 29960617 10.1016/j.cjca.2018.04.033

[40] Hoppe EJ, Main WP, Kelley SR et al (2017) Urinary retention following colorectal surgery. Am Surg 83:3–7 28234104

[41] Yao Q, Sun Q-N, Zhou J-J et al (2023) Robotic-assisted intracorporeal versus extracorporeal techniques in sigmoidectomy: a propensity score-matched analysis. J Robot Surg 17:2479–2485 37515681 10.1007/s11701-023-01678-9

[42] Zhuang C-L, Ye X-Z, Zhang C-J et al (2013) Early versus traditional postoperative oral feeding in patients undergoing elective colorectal surgery: a meta-analysis of randomized clinical trials. Dig Surg 30:225–232 23838894 10.1159/000353136

[43] Chasserant P, Gosgnach M (2016) Improvement of peri-operative patient management to enable outpatient colectomy. J Visc Surg 153:333–337 27671006 10.1016/j.jviscsurg.2016.07.006

[44] Asao T, Kuwano H, Nakamura J et al (2002) Gum chewing enhances early recovery from postoperative ileus after laparoscopic colectomy. J Am Coll Surg 195:30–32 12113542 10.1016/s1072-7515(02)01179-1

[45] Schuster R, Grewal N, Greaney GC, Waxman K (2006) Gum chewing reduces ileus after elective open sigmoid colectomy. Arch Surg 141:174–176 16490895 10.1001/archsurg.141.2.174

[46] Wise J (2022) Persistent understaffing of the NHS is putting patients at risk, say MPs. BMJ. 10.1136/bmj.o1866 35882403 10.1136/bmj.o1866

[47] Khan S, Mian A (2020) Papering over the cracks in the NHS. Int J Health Policy Manag. 10.34172/ijhpm.2020.160 10.34172/ijhpm.2020.160 PMC9278597 32861234

